# Noninvasive Assessment of Vascular Endothelial Growth Factor and Prognosis in Gastric Cancer Through Radiomic Features

**DOI:** 10.14309/ctg.0000000000000802

**Published:** 2024-12-30

**Authors:** Hao Feng, Kangneng Zhou, Qingyu Yuan, Zhiwei Liu, Taojun Zhang, Hao Chen, Benjamin Xu, Zepang Sun, Zhen Han, Hao Liu, Shitong Yu, Tao Chen, Guoxin Li, Wenlan Zhou, Jiang Yu, Weicai Huang, Yuming Jiang

**Affiliations:** 1 Department of General Surgery & Guangdong Provincial Key Laboratory of Precision Medicine for Gastrointestinal Tumor, Nanfang Hospital, Southern Medical University, Guangzhou, China;; 2 College of Computer Science, Nankai University, Tianjin, China;; 3 Department of Medical Imaging Center, Nanfang Hospital, Southern Medical University, Guangzhou, China;; 4 Department of PET Center, Nanfang Hospital, Southern Medical University, Guangzhou, China;; 5 Lynbrook High School, San Jose, California, USA;; 6 Beijing Tsinghua Changgung Hospital, School of Clinical Medicine, Tsinghua University, Beijing, China;; 7 Department of Gastrointestinal Surgery, the First Affiliated Hospital of Guangzhou Medical University, Guangzhou, China;; 8 Department of Radiation Oncology, Wake Forest University School of Medicine, Winston-Salem, North Carolina, USA.

**Keywords:** gastric cancer, VEGF, radiomics, noninvasive prediction, survival prognosis, [18F] FDG PET/CT

## Abstract

**INTRODUCTION:**

Gastric cancer (GC) is a leading cause of cancer-related deaths worldwide, with delayed diagnosis often limiting effective treatment options. This study introduces a novel, noninvasive radiomics-based approach using [18F] FDG PET/CT (fluorodeoxyglucose positron emission tomography/computed tomography) to predict vascular endothelial growth factor (VEGF) status and survival in patients with GC. The ability to noninvasively assess these parameters can significantly influence therapeutic decisions and outcomes.

**METHODS:**

We conducted a retrospective study involving patients diagnosed with GC, stratified into training, validation, and test groups. Each patient underwent a [18F] FDG PET/CT scan, and radiomic features were extracted using dedicated software. A Radiomics Score (RS) was calculated, serving as a predictor for VEGF status. Statistical analyses included logistic regression and Cox proportional hazards models to evaluate the predictive power of RS on survival outcomes.

**RESULTS:**

The developed radiomics model demonstrated high predictive accuracy, with the RS formula achieving an area under the receiver operating characteristic curve of 0.861 in the training cohort and 0.857 in the validation cohort for predicting VEGF status. The model also identified RS as an independent prognostic factor for survival, where higher RS values correlated with poorer survival rates.

**DISCUSSION:**

The findings underscore the potential of [18F] FDG PET/CT radiomics in transforming the management of GC by providing a noninvasive means to assess tumor aggressiveness and prognosis through VEGF status. This model could facilitate earlier and more tailored therapeutic interventions, potentially improving survival outcomes in a disease marked by typically late diagnosis and limited treatment success.

## INTRODUCTION

Gastric cancer (GC) ranks as the fifth most common cancer globally and is the fourth leading cause of cancer-related deaths (1). Although surgical resection remains the primary curative method for patients with early-stage GC, the lack of early symptoms, particularly in China, often leads to late-stage diagnosis for most patients (2–4). It underscores the importance of perioperative or adjuvant chemotherapy (5,6). Studies indicate that chemotherapy can improve the survival rates of patients with GC, yet the degree of improvement in survival tends to be limited for many advanced-stage patients (7–9). Consequently, targeted therapies have become widely used in treating patients with advanced-stage GC (10–12).

The overexpression of vascular endothelial growth factor (VEGF) in GC has been extensively documented (13). VEGF, produced by tumor cells and the surrounding stromal tissue, stimulates endothelial cell proliferation and survival and enhances vascular permeability, thus promoting neovascularization and exacerbating tumor growth and metastasis (14–17). Furthermore, VEGF can also influence tumor behavior by modulating vascular function or the tumor's immune microenvironment (18–21). The tissue-based VEGF status and serum VEGF levels are associated with the late stages of the disease and poor prognosis (22). Although antiangiogenic drugs such as ramucirumab and apatinib have been approved for treating advanced or metastatic GC in China, showing potential to prolong overall patient survival, in clinical practice, these drugs are often used without considering the VEGF status (23–28). This approach may not fully exploit the potential effects of the drugs, as studies indicate that the level of VEGF expression is related to patient responsiveness to these treatments (29–31). Thus, dynamically assessing VEGF status is crucial for further optimizing the clinical benefits of anti-VEGF drugs, especially in developing personalized treatment plans and improving efficacy (32,33). However, the current gold standard for evaluating VEGF status remains invasive, often requiring collecting and analyzing biological specimens, such as tumor tissue (34–36). This approach not only imposes additional physical burdens on patients but also may carry higher risks and technical difficulties in certain situations, such as late-stage disease or poor clinical conditions (37,38). These challenges underscore the importance of developing a noninvasive, rapid, and reliable method to assess VEGF status to safely and effectively implement targeted treatment strategies, optimize efficacy, and alleviate patient treatment burden.

Radiomics, as an emerging field, uses advanced algorithms and statistical analysis tools to quantitatively analyze tumor characteristics through high-throughput radiomic features, providing a novel, noninvasive approach for tumor detection, prognostic assessment, and subtype classification (39). This field integrates the latest medical imaging technology and computer science advancements, enabling researchers to extract unprecedented biological information from routine medical images (40–42). Particularly, in highly heterogeneous tumors such as GC, the application of radiomics holds significant potential (43–45). By analyzing radiomics features from [18F] fluorodeoxyglucose positron emission tomography/computed tomography (FDG PET/CT) images, not only can various metabolic phenotypes associated with different gene mutations be revealed but also microscopic pathological changes can be detected, which may directly correlate with the expression status of VEGF (46–49). Furthermore, radiomics can offer profound insights into the tumor microenvironment, such as tumor blood flow dynamics, cell density, and the heterogeneity of tissue structure, details that are challenging to observe with traditional imaging techniques.

The aim of this study was to establish a noninvasive model predicting VEGF status and survival prognosis of patients with GC by analyzing [18F] FDG PET/CT radiomics features. The development of this model will enable clinicians to tailor more precise treatment plans based on the tumor's radiomics characteristics, particularly when selecting anti-VEGF treatment strategies. This approach not only facilitates personalized medication selection and treatment planning for physicians but also has the potential to significantly enhance the targeting and efficacy of therapies. Moreover, validating this predictive model may improve treatment outcomes and quality of life of patients with GC, providing a scientific basis for personalized health care. Through this model, early identification of treatment-responsive patients can be achieved, optimizing treatment pathways and reducing unnecessary side effects and health care costs, ultimately enhancing patients' overall treatment experience and quality of life.

## METHODS

### Patient selection and data collection

This retrospective analysis obtained approval from the Institutional Review Board of the Southern Medical University Nanfang Hospital. All patients participating in the study provided written informed consent before the study initiation. Inclusion criteria comprised (i) patients confirmed with primary GC through biopsy or pathological tissue verification, (ii) all patients underwent examination under the same PET/CT scanning conditions, (iii) patients had complete clinical and pathological data along with available follow-up data, and (iv) patients did not receive any form of pretreatment before the PET/CT scan. Exclusion criteria included (i) patients who received neoadjuvant radiotherapy, neoadjuvant chemotherapy, or neoadjuvant chemo-radiotherapy before the PET/CT scan; (ii) patients with poor quality PET/CT images; and (iii) patients unable to provide complete clinical and pathological data or follow-up information.

**Figure 1.**
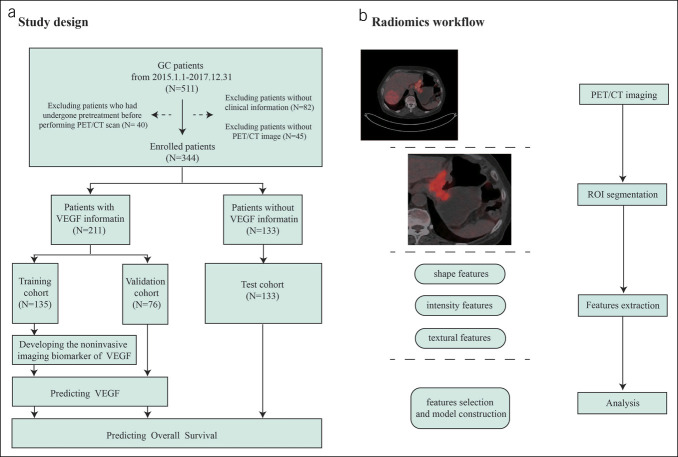
Study flowchart for predicting VEGF status and overall survival and the radiomics workflow. (**a**) Flowchart of the study population; (**b**) Workflow for developing the PET/CT radiomics model. CT, computed tomography; GC, gastric cancer; PET, positron emission tomography; ROI, region of interest; VEGF, vascular endothelial growth factor.

This study included patients confirmed with primary GC through biopsy or surgical pathology. Based on the pathological findings, patients were categorized into VEGF(+) and VEGF(−) groups. VEGF(+) patients were randomly allocated in approximately a 2:1 ratio to the training, and validation sets for model development and initial validation; patients lacking VEGF status were assigned to the testing set for the final model evaluation (Figure 1a). All patients underwent the same PET/CT examination, with image evaluation performed by 2 experienced radiologists. In most cases, the evaluation results of the 2 doctors remained consistent. In the event of any disagreement, the doctors reached a consensus through discussion and consultation, ensuring the accuracy and consistency of the evaluations. As disagreements were rare and could be effectively resolved through consultation, a third doctor was not introduced as arbitrators. The high level of agreement between the 2 doctors provided strong assurance for the reliability of the analytical results. The image workflow is illustrated in Figure 1b. Details of the PET/CT scanning and image processing are presented in the Supplementary Materials (http://links.lww.com/CTG/B242).

Each patient's baseline information, including age, gender, tumor size, tumor differentiation, tumor location, and levels of biomarkers, was recorded. All patients were staged for tumors, lymph nodes, and distant metastasis, according to the American Joint Committee on Cancer's eighth edition TNM staging manual. Patient follow-up included regular clinical assessments and imaging follow-ups to monitor disease progression and treatment response. Overall survival (OS) was defined as the period from the date of diagnosis to death from any cause.

### Subgroup analysis of patients with stage 3–4 and tumor size ≥4 cm

To further evaluate the performance of the Radiomics Score (RS) model in advanced-stage patients, we conducted a separate analysis of patients with stage 3–4 disease and tumor size **≥**4 cm. These patients were selected from the training, validation, and test cohorts, meeting the inclusion criteria outlined above and the quality requirements for PET/CT scans. In this subgroup, we used receiver operating characteristic (ROC) curves to assess the model's performance in predicting VEGF status and calculated the area under the curve (AUC) and 95% confidence interval [CI]. To further evaluate the prognostic value of the model, we plotted survival curves using the Kaplan-Meier method and compared the survival differences between the RS-high and RS-low groups using the log-rank test. In addition, we performed a stratified analysis based on whether chemotherapy was administered to explore the impact of chemotherapy on patients in different risk groups.

### Constructing noninvasive imaging biomarkers

This study used the Least Absolute Shrinkage and Selection Operator (LASSO) regression model to identify predictive features most relevant to the expression status of VEGF from a wide array of features. The LASSO regression effectively dealt with multicollinearity issues in feature selection by applying penalty terms to the coefficients and determining the optimal penalty coefficient through cross-validation. Based on the results of the LASSO regression, a RS was developed, which integrated several key features to predict the VEGF status. This score was calibrated by training data from a cohort of patients, and a threshold was established to distinguish between high and low VEGF expression states within the patient population. Subsequently, using this threshold, the patients were divided into 2 groups and survival analysis was performed on each group (Tables S1 and S2, http://links.lww.com/CTG/B243).

### Developing predictive models

Univariate logistic regression or Cox regression analyses were used in the training cohort to examine various clinical pathological variables to identify potential predictive factors for VEGF status and OS of patients with GC. Subsequently, variables showing statistically significant differences (*P* < 0.05) were selected for further multivariate logistic regression or Cox regression analysis. The likelihood ratio test served as the termination rule for implementing backward stepwise selection (Table S3, http://links.lww.com/CTG/B243). Variables that remained statistically significant (*P* < 0.05) in the multivariate logistic regression or Cox regression analyses were included in the nomograms for predicting VEGF status or OS. Validation and clinical utility of the noninvasive imaging biomarkers are provided in the Supplementary Materials (http://links.lww.com/CTG/B242).

### Statistical analysis

All statistical analyses in this study were conducted using SPSS version 21.0 (IBM) and R version 4.0.2. Two-tailed *t*-tests were used for analyzing continuous variables, while χ^2^ tests or Fisher exact tests were used for categorical variables. Univariate and multivariate binary logistic regression analyses or Cox regression analyses were performed to evaluate the variables predicting VEGF status or OS. A 2-sided *P* value of <0.05 was considered statistically significant.

In the R environment, various specialized packages were used to enhance the accuracy of the radiomics feature selection and the interpretability of the predictive model. Initially, the Minimum Redundancy Maximum Relevance (mRMR) algorithm from the “more” package was used to eliminate redundancy and irrelevant features within the radiomics data set, ensuring that the selected features were not only statistically associated with VEGF status but also independent of each other. The LASSO logistic regression method, implemented through the “glmnet” package, was used for feature selection to further reduce model complexity and enhance its generalizability. The “pROC” package was used to plot ROC curves and calculate AUC values to assess the predictive accuracy and calibration of the model. In addition, the “pec” package was used to generate calibration plots, aiding in evaluating the consistency between the predicted probabilities of the model and actual observed outcomes. Furthermore, the “rms” package was used to construct nomograms for personalized prognoses based on the model predictions. For the subgroup of patients with stage 3–4 disease and tumor size ≥ 4 cm, we similarly used the “pROC” package to calculate the AUC and its 95% CI for both the training and validation groups. Kaplan-Meier survival curves were plotted using the “survminer” package. We used the log-rank test to compare survival differences between the RS-high and RS-low groups and further performed a stratified analysis based on whether chemotherapy was administered to explore its impact on survival across different risk groups. Finally, detailed Kaplan-Meier survival curves and decision curve analysis were generated using the “survminer” and “ggDCA” packages, illustrating the clinical utility of the model at various clinical decision thresholds.

### Ethical statement

This study was approved by the Clinical Ethics Committee of the institution with reference number NFEC-2017-171.

## RESULTS

### Correlation analysis between clinical pathological features and VEGF status

This study included 344 patients with GC who underwent treatment at our institution between January 1, 2015, and December 31, 2017, with detailed patient information shown in Figure 1a. Clinical pathological features are presented in Table S1 (http://links.lww.com/CTG/B243). Among them, VEGF status was available for 211 patients, divided into a training cohort (135 patients) and a validation cohort (76 patients), while the remaining 133 patients without VEGF status determination comprised the test cohort. We compared the baseline characteristics of patients in the training and validation cohorts to ensure the comparability of the 2 groups across key clinical variables, including age, sex, tumor size, differentiation, tumor location, T stage, N stage, and M stage (Table S4, http://links.lww.com/CTG/B243). The results showed that the *P* values for all variables between the training and validation groups were greater than 0.05, indicating no significant differences in most baseline characteristics between the 2 groups, thereby ensuring the reliability of model development and validation. Analysis of the training and validation cohorts revealed significant differences between VEGF(+) and VEGF(−) patients about M stage and tumor size (*P* < 0.05), indicating a statistical correlation between VEGF status and these clinical pathological features.

Furthermore, in the training cohort, VEGF(+) and VEGF(−) patients also exhibited significant differences in tumor location and T stage (*P* < 0.05), further emphasizing the connection between VEGF expression and tumor behavior. The intraclass correlation coefficients of radiomics features extracted independently by 2 radiologists exceeded 0.75, demonstrating high consistency. Consequently, all subsequent analyses were based on the features extracted by the first radiologist to ensure the accuracy and consistency of the analysis. These findings confirm a significant association between VEGF status and key clinical pathological features in patients with GC.

In conclusion, this study analyzed data from 344 patients with GC, revealing a significant correlation between VEGF status and clinical pathological features, such as M stage, tumor size, location, and T stage. This highlights the potential value of VEGF as a prognostic biomarker for GC. The consistency of genomic features ensures the reliability of the analysis results.

### Noninvasive imaging predicts VEGF status through RS

In this study, we used the mRMR algorithm to eliminate redundant features, selecting 8 CT and 6 PET features. With these features, we conducted LASSO regression analysis to develop a noninvasive imaging biomarker, termed RS, for predicting the status of VEGF. This scoring model's specific formula and graphical representation are presented in the supplementary materials (http://links.lww.com/CTG/B242) and Figure S1a,b (http://links.lww.com/CTG/B237). In both the training and validation cohorts, significant differences were observed in RS between VEGF(+) and VEGF(−) patients (*P* < 0.01), indicating the ability of this score to effectively differentiate patients based on VEGF status (Table S1 [http://links.lww.com/CTG/B243],). Univariate analysis revealed a significant association between VEGF status and various clinical pathological features, such as tumor size, tumor location, T stage, M stage, and RS (Table S2, http://links.lww.com/CTG/B243). These variables were further examined using multivariate logistic regression analysis, demonstrating that RS is a reliable, independent predictor of VEGF status (Odds Ratio: 9.009, 95% CI 3.506–23.151, *P* < 0.001) (Table S3, http://links.lww.com/CTG/B243). Based on these findings, a nomogram integrating RS, tumor location, and M stage was developed to predict VEGF status in the training cohort (Figure 3).

**Figure 2.**
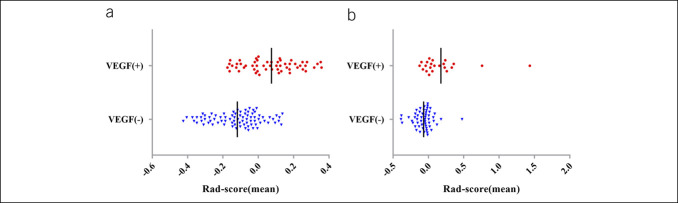
Distribution of RS based on VEGF status. (**a** and **b**) RS for each patient and the median values for each cohort. (**a**) Training cohort; (**b**) validation cohort. VEGF(+) indicates positive VEGF status; VEGF(−) indicates negative VEGF status. RS, Radiomics Score; VEGF, vascular endothelial growth factor.

**Figure 3.**
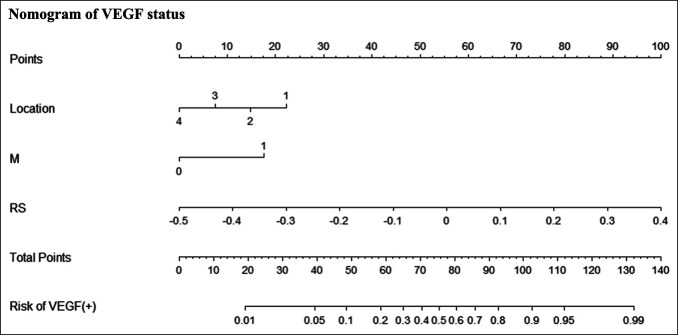
Noninvasive biomarkers for VEGF status in patients with gastric cancer. Locate each variable for the patient on the variable-score axis. Draw a vertical line from the point on the coordinate axis to determine the contribution of the patient's score to the VEGF risk probability. Repeat this process for each variable. Sum the scores of all-risk factors. Position the final total on the total points axis and draw a line to indicate the patient's probability of VEGF status. Locations on pathological specimens are denoted as 1: Cardia, 2: Body, 3: Anus, 4: Entire. M: Clinical M stage, 1: Metastasis, 0: None. RS: VEGF RS. RS, Radiomics Score; VEGF, vascular endothelial growth factor.

**Figure 4.**
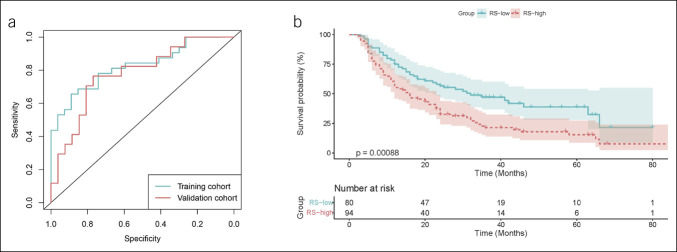
VEGF status prediction and survival analysis of patients with stage 3–4 and tumor size > 4 cm using the RS model. (**a**) Receiver operating characteristic curves for VEGF status prediction in patients with stage 3–4 and tumor size > 4 cm using the RS model. (**b**) Kaplan-Meier survival analysis of patients with stage 3–4 and tumor size >4 cm. RS, Radiomics Score; VEGF, vascular endothelial growth factor.

To further evaluate the performance of the RS model in advanced-stage patients, we conducted a separate analysis of patients with stage 3–4 disease and tumor size >4 cm. We plotted ROC curves for the training and validation cohorts in this subgroup. The AUC for the training cohort was 0.810 (95% CI: 0.699–0.921), and the AUC for the validation cohort was 0.778 (95% CI: 0.636–0.920), indicating that the RS model maintained good predictive performance for VEGF status in these patients (Figure 4a). In addition, in the training, validation, and test cohorts, the Kaplan-Meier survival analysis was performed for patients with stage 3–4 disease and tumor size >4 cm. The results showed that the survival rate of the RS-high group was significantly lower than that of the RS-low group (*P* < 0.001), regardless of whether chemotherapy was administered (Figure 4b).

In summary, this study developed an RS based on CT and PET images using the mRMR algorithm and LASSO regression analysis to effectively predict the VEGF status in patients with GC. The RS demonstrated high predictive accuracy and was confirmed as an independent predictor of VEGF status. Furthermore, the nomogram based on RS enhanced the precision and utility of predictions, highlighting its significant potential in diagnosing and treating GC.

### Validation and assessment of a noninvasive imaging biomarker for VEGF

In the training cohort, the AUC for RS predicting VEGF status was 0.819 (95% CI, 0.743–0.880), surpassing the predictive performance of clinical features (such as M stage and tumor location), demonstrating the predictive value of the imaging biomarker (Figure S2a [http://links.lww.com/CTG/B238] and Table S5 [http://links.lww.com/CTG/B243]). Moreover, the nomogram integrating RS, M stage, and tumor location exhibited higher positive accuracy in predicting the VEGF status of GC compared with individual factors, with an AUC of 0.861 (95% CI, 0.791–0.915) in the training cohort (Figure S2a [http://links.lww.com/CTG/B238] and Table S5 [http://links.lww.com/CTG/B243]). Similar results were observed in the validation cohort (Figure S2b [http://links.lww.com/CTG/B238] and Table S5 [http://links.lww.com/CTG/B243]). Subsequently, by determining the critical value of the Youden index (Table S6, http://links.lww.com/CTG/B243), patients were stratified into high and low RS groups based on the optimal RS threshold of −0.0137. In both the training and validation cohorts, RS demonstrated strong effectiveness in observed sensitivity, specificity, and accuracy (Table S6, http://links.lww.com/CTG/B243). Furthermore, the calibration curves of RS in both cohorts exhibited good consistency between predicted and observed outcomes (Figure S2c,d, http://links.lww.com/CTG/B238). Decision curve analysis revealed that the nomogram predicting VEGF status provided greater clinical utility than considering only M stage and tumor location or adopting all treatment or no treatment strategies (Figure S2e,f, http://links.lww.com/CTG/B238).

In conclusion, this study demonstrates the efficacy of RS derived from CT and PET images in predicting the VEGF status in patients with GC. RS exhibited superior predictive capability compared with traditional clinical features in both the training and validation cohorts, with significant AUC values. In addition, integrating RS with other clinical factors in a nomogram enhanced prediction accuracy. The high sensitivity, specificity, and calibration of RS underscore its effectiveness and practicality as a noninvasive imaging biomarker. These results highlight the potential utility of RS scoring in managing GC.

### The predictive role of RS in OS of patients with GC

**Figure 5.**
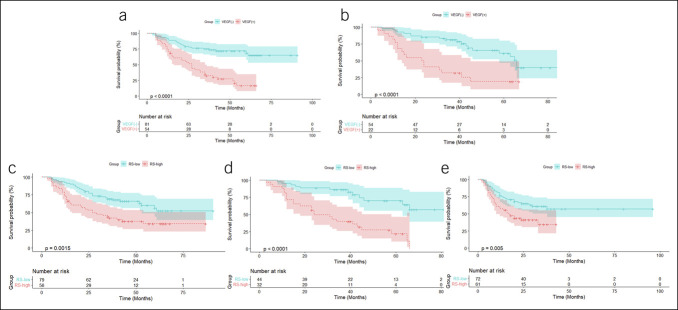
Kaplan-Meier analysis of GC patients in training, validation, and testing groups. (**a**) Total survival rates of VEGF-positive and VEGF-negative patients with GC in the training (**a**) and validation (**b**) groups, stratified by pathological classification. (**c**–**e**) Total survival rates of patients with GC in different risk groups (RS-high or RS-low) after stratification by RS in the training (**c**), validation (**d**), and testing (**e**) cohorts. GC, gastric cancer; RS, Radiomics Score; VEGF, vascular endothelial growth factor.

GC patients with a cheerful expression of VEGF typically have a poorer prognosis (22), a finding validated in both the training and validation cohorts (, ). Statistically, the survival rate of VEGF-positive patients with GC is lower than that of VEGF-negative patients (*P* < 0.0001;,). Survival curves of the high and low RS groups were analyzed in the training, validation, and testing cohorts to determine the predictability of RS in the survival cohorts. Interestingly, significant differences in OS were observed between the RS-high and RS-low groups in each cohort (*P* < 0.001; Figure 5c–e). In the training cohort, the 3-year and 5-year OS rates for the RS-low group (67.8% and 56.3%, respectively) were significantly higher than those for the RS-high group (42.1% and 34.2%, respectively). In the validation cohort, the 3-year and 5-year OS rates for the RS-low group were 86.3% and 70.0%, respectively, while those for the RS-high group were 39.7% and 21.8%, respectively. A similar trend was observed in the testing cohort, where the 3-year OS rate for the RS-low group was 60.8% compared with 47.3% for the RS-high group. Stratification of patients with GC into RS-high and RS-low groups based on whether they received chemotherapy was performed to assess the effect of chemotherapy on these cohorts. Interestingly, regardless of chemotherapy status, patients in the RS-high group consistently exhibited poorer OS than those in the RS-low group (*P* < 0.05; Figure S3, http://links.lww.com/CTG/B239).

Furthermore, univariate Cox regression analysis was conducted to explore the relationship between clinical pathological factors and OS. In the univariate analysis, factors such as tumor size, T stage, and N stage demonstrated significant impacts on survival, particularly advanced T stage (T3-4) with a hazard ratio (HR) of 6.242, indicating a substantial reduction in survival with higher T stages, significance level less than 0.001 (Table 1). In the multivariate analysis, RS and M stages were identified as independent key predictors of survival in patients with GC. The HR for RS was 5.063 (95% CI: 1.018–25.176, *P* = 0.047), underscoring its strength as an independent predictive factor, while the HR for the M stage was 5.051 (95% CI: 2.889–8.834, *P* < 0.001), highlighting the significant impact of cancer metastasis status on survival (Table 1). Similar results were observed in the validation and test cohorts (Tables S7 and S8, http://links.lww.com/CTG/B243). Consequently, a nomogram predicting OS was constructed based on RS in the training cohort (Figure S4, http://links.lww.com/CTG/B240).

**Table 1.** Univariate and multivariable Cox regression analysis of the noninvasive biomarker predicts overall survival in the training cohort

Variable	Univariable			Multivariable		
	HR	95% CI	*P*	HR	95% CI	*P*
Gender (female vs male)	1.343	0.817–2.208	0.245	\	\	\
Age (≥60 vs <60 yr)	1.263	0.776–2.058	0.348	\	\	\
CEA (elevate vs normal)	1.662	0.716–3.859	0.237	\	\	\
CA19-9 (elevate vs normal)	2.019	0.915–4.453	0.082	\	\	\
Differentiation						
Well	Reference	\	\	\	\	\
Moderate	1.736	0.209–14.443	0.610	\	\	\
Poor or undifferentiation	4.613	0.638–33.353	0.130	\	\	\
Size(≥4 vs <4 cm)	2.858	1.724–4.741	<0.001*	1.309	0.700–2.448	0.400
Location						
Cardia	Reference	\	\	\	\	\
Body	1.233	0.564–2.694	0.599	\	\	\
Antrum	0.762	0.413–1.405	0.384	\	\	\
Whole	1.964	0.937–4.117	0.074	\	\	\
T stage						
T1-2	Reference	\	\	Reference	\	\
T3-4	6.242	2.500–15.588	<0.001*	2.272	0.833–6.200	0.109
N stage						
N0	Reference	\	\	Reference	\	\
N1	0.540	0.208–1.402	0.206	0.617	0.233–1.636	0.332
N2	1.771	0.745–4.209	0.196	1.246	0.506–3.067	0.633
N3a	2.671	1.158–6.161	0.021*	1.871	0.755–4.636	0.176
N3b	2.359	1.077–5.168	0.032*	1.163	0.475–2.850	0.741
M stage	7.309	4.355–12.265	<0.001*	5.051	2.889–8.834	<0.001*
RS	32.258	7.147–145.595	<0.001*	5.063	1.018–25.176	0.047*

95%. CI, 95% confidence interval; CA19-9, carbohydrate antigen 19-9; CEA, carcinoembryonic antigen; HR, hazard ratio; M stage, clinical M stage; N stage, clinical N stage; RS, vascular endothelial growth factor Rad-score; T stage, clinical T stage.

* Significant difference.

This study demonstrates that RS is an effective tool for predicting OS in patients with GC. The results indicate that regardless of chemotherapy treatment, patients in the high RS group have significantly lower survival rates compared with those in the low RS group. Furthermore, RS is significantly associated with OS in univariate analysis and confirmed as an independent predictor of OS in multivariate analysis.

### Prognostic value of the nomogram for survival prediction in patients with GC

The predictive performance of the nomogram for surgical timing in patients with GC was evaluated through time-dependent ROC analysis at various follow-up intervals in each cohort. Particularly notable was the outstanding performance of the nomogram in the training cohort, with the AUC reaching 0.875 and 0.866 for predicting survival at 1, 2, and 3 years, respectively, indicating its high accuracy in forecasting patients' short-term and mid-term survival rates. This high level of precision reflects the robust predictive capability of the model and its potential clinical utility (Figure S5a, http://links.lww.com/CTG/B241). In the validation cohort, although the AUC values experienced a slight decrease to 0.773, 0.834, and 0.831 for the respective follow-up periods, it still demonstrated good predictive performance across different sample sets, showcasing the model's strong generalizability (Figure S5b, http://links.lww.com/CTG/B241). Similar results were observed in the test cohort, with AUC values of 0.806, 0.831, and 0.818 for 1-year, 2-year, and 3-year survival predictions, further confirming the stability and reliability of the model on unseen data (Figure S5c, http://links.lww.com/CTG/B241).

Moreover, data in Table S9 (http://links.lww.com/CTG/B243) provide a more comprehensive assessment perspective, comparing the performance of the nomogram with solely clinical M staging in predicting OS. The nomogram exhibited a higher C-index of 0.783 in the training cohort compared with the C-index of 0.737 with clinical M staging alone, indicating that the nomogram, constructed based on a combination of various clinical and radiomics features, can more accurately predict survival rates (Table S9, http://links.lww.com/CTG/B243). In the validation and test cohorts, the C-index of nomogram was 0.757 and 0.764, respectively, surpassing the C-index of 0.656 and 0.691 when using M staging alone, highlighting the nomogram's consistent outperformance of traditional models in predictive accuracy and clinical applicability across different data sets.

These analyses show that the nomogram for surgical timing significantly outperforms the traditional clinical M staging method statistically and demonstrates high accuracy and consistency in practical clinical use, making it a powerful tool for evaluating survival predictions in patients with GC.

## DISCUSSION

Radiomics is an emerging field that integrates medical imaging with computer technology. Extracting high-dimensional quantitative features such as tumor shape, size, texture, and density from conventional medical images offers a new perspective for early diagnosis, treatment assessment, and prognosis evaluation of GC (39). GC, being one of the most common malignant tumors globally, directly affects patient survival rates and quality of life through its early diagnosis and treatment outcomes (50). Radiomics provides a fresh outlook on GC's early diagnosis, treatment assessment, and prognosis evaluation (39). This technology can unveil tumor biology and heterogeneity, enhance diagnostic accuracy, and assist in developing more targeted treatment plans. Particularly beneficial for late-stage patients who are unsuitable for surgery, Radiomics offers a noninvasive assessment method, significantly optimizing the practice of personalized medicine (51).

Early studies in GC research focused on exploring VEGF as a potential therapeutic target due to its crucial role in promoting tumor angiogenesis (52–54). Numerous studies have concentrated on assessing VEGF expression levels through blood and tissue samples, investigating the correlations of these levels with disease severity, treatment response, and prognosis (55,56). For instance, techniques such as ELISA or immunohistochemistry have been used to directly measure VEGF levels in tumor tissues or serum (57–59). However, these methods are often invasive and may not be feasible for advanced-stage patients who are not candidates for surgery. By contrast, this study introduces a novel approach using radiomics features from [18F] FDG PET/CT scans to predict the VEGF status of GC patients. The advantage of this method lies in its noninvasiveness, ability for continuous monitoring, and applicability to patients at all stages of the disease, including those ineligible for surgery in late stages.

In this study, we successfully identified radiomics features significantly associated with the expression status of VEGF by analyzing PET/CT images, including tumor metabolic activity, heterogeneity, and morphological variations. These features are closely linked to the biological characteristics reported in existing literature, but our study further provides new insights by quantifying these image features. Although VEGF is generally correlated with tumor invasiveness and poor prognosis in past research, few studies have evaluated its expression through noninvasive imaging modalities. Therefore, the methodological innovation in this study offers a new perspective for understanding the role of VEGF in the progression of GC.

In this study, the calculation formula for RS was designed based on multivariable analysis, which sharply contrasts with traditional univariate analysis methods. The RS formula demonstrated strong predictive capability and reproducibility by comparing data between the training and validation groups. Furthermore, the RS calculation considers single image features and integrates multiple radiomics features, a less common practice in prior research. This comprehensive assessment approach better captures tumors' biological behavior, enhancing the predictive model's accuracy and utility.

In this study, ROC curve analysis was used to evaluate the effectiveness of the RS in predicting the VEGF status. By carefully examining the AUC values in the training and validation groups, we confirmed the stability and reliability of the model across different sample sets. Compared with previous studies, the ROC analysis in our research validated the model's predictive capability and underscored the importance of adjusting thresholds to optimize sensitivity and specificity in practical clinical applications, providing direct guidance for clinical decision-making.

An important discovery of this study is that the RS can not only predict the VEGF status but also independently predict the survival period of patients with GC, providing a theoretical basis for the clinical application of RS. Although prior studies have seen attempts by researchers to predict the prognosis of GC using various biomarkers, few have offered such direct and specific prognostic information noninvasively. The results of this study demonstrate that the RS model maintains good predictive performance in advanced-stage patients with stage 3–4 disease and tumor size ≥4 cm, supporting prognosis assessment and personalized treatment strategies for high-risk patients. Using RS as a biomarker derived from imaging data significantly expands its clinical utility, especially in customizing individualized treatment plans (60–62).

This study further confirms that VEGF-positive patients have a worse prognosis, aligning with findings from previous biomedical research. However, the unique aspect of this study lies in using the noninvasive imaging biomarker RS for this prediction. This approach enhances safety and convenience in assessment, and it may also facilitate future clinical practice by enabling this assessment through routine PET/CT scans, thus avoiding the need for additional invasive procedures for patients.

Although this study provides new insights into predicting VEGF status in GC using radiomics, several significant limitations cannot be overlooked. First, although patients were selected by excluding those who had undergone pretreatment before PET/CT, we were unable to comprehensively rule out other factors that may influence VEGF levels, such as chronic inflammation, autoimmune diseases, infections, or the use of specific medications, which could have affected the accuracy of the study results to some extent. Second, due to its retrospective design and data sourced from a single center, the generalizability and applicability of the results may be compromised. The relatively small sample size, particularly in advanced statistical analyses, could affect the stability and reliability of certain conclusions. In addition, limitations in technology and operations may influence the extraction and analysis of radiomics features, potentially introducing heterogeneity in results across different institutions. Last, the study's failure to encompass all potential clinical variables, such as specific treatment regimens and responses, could affect the prediction of VEGF status and survival analysis.

Future research in this field should strive to overcome limitations and expand in several key areas. First, prospective, multicenter study designs should be considered to increase sample size and diversity, thus enhancing the generalizability and reproducibility of research findings. Second, there is a need to further optimize the image analysis techniques of radiology, developing more precise algorithms to manage and analyze large sets of image data to ensure accurate and consistent extraction of radiology features. Future research should focus on eliminating confounding factors affecting VEGF levels, such as chronic inflammation and medication use, to ensure more robust data and reliable results. In addition, integrating radiomics with other modalities such as genomics and proteomics can offer comprehensive biomarker information for more accurate disease progression and treatment response predictions. Last, research should explore the application of radiology in clinical decision support systems, particularly in the context of personalized medicine and precision therapy, and understand how to effectively use radiology information to guide the selection and adjustment of treatment strategies for GC. These efforts are expected to drive the advancement of precision medicine, ultimately improving treatment outcomes and quality of life for patients with GC.

**Figure 6.**
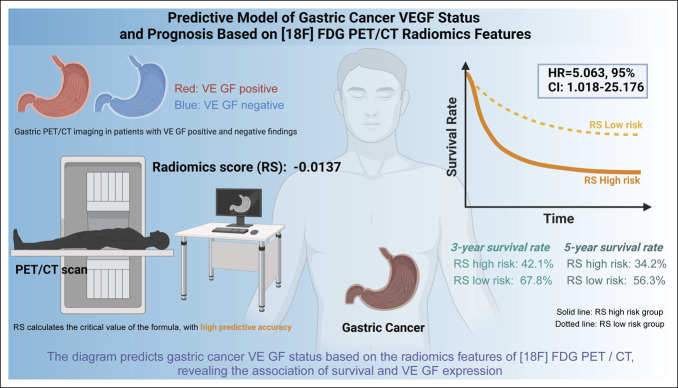
Predictive model of gastric cancer VEGF status and prognosis based on [18F] FDG PET/CT radiomics features. CT, computed tomography; FDG, fluorodeoxyglucose; HR, hazard ratio; PET, positron emission tomography; VEGF, vascular endothelial growth factor

This study successfully established a noninvasive model for predicting VEGF status and prognosis of GC patients using [18F] FDG PET/CT radiomics analysis (Figure 6). The study results demonstrate that the RS can effectively predict the VEGF status. The formula and threshold value of RS obtained through calculation can be predictive tools. RS has been proven to be an independent prognostic factor for OS in patients with GC, indicating its high predictive value, especially in predicting the survival prognosis of VEGF-positive patients. This finding underscores the significant clinical relevance of RS in predicting patient outcomes.

## CONFLICTS OF INTEREST

**Guarantor of the article:** Yuming Jiang, MD.

**Specific author contributions:** H.F., K.Z., and Q.Y. contributed equally to the study design, data acquisition, and initial analysis of radiomics features. Z.L., T.Z., H.C., and B.X. assisted in data processing and the development of the Radiomics Score. Z.S., Z.H., H.L., S.Y., and T.C. participated in clinical data collection and imaging interpretation. G.L. and W.Z. provided expertise in PET/CT analysis and contributed to methodological supervision. J.Y., W.H., and Y.J. coordinated the research, provided critical revisions, and oversaw all project stages. W.Z., J.Y., W.H., and Y.J. supervised the study and finalized the manuscript.

**Financial support:** This study was supported by National Natural Science Foundation of China (82102156, 82302301), China Postdoctoral Science Foundation (2023M731567), Guangdong Provincial Key Laboratory of Precision Medicine for Gastrointestinal Cancer (2020B121201004), the Key-Area Research and Development Program of Guangdong Province (2021B0101420005), Natural Science Foundation of Guangdong Province (2021A1515011146, 2023A1515010785), the Guangdong Provincial Major Talents Project (No. 2019JC05Y361).

**Potential competing interests:** None to report.

**Data availability statement:** The original contributions presented in the study are included in the article/supplementary materials (http://links.lww.com/CTG/B242), further inquiries can be directed to the corresponding author.

**Study Highlights**

**WHAT IS KNOWN**

✓ Vascular endothelial growth factor (VEGF) is overexpressed in gastric cancer (GC) and associated with poor prognosis.
✓ As a heterogeneous disease, GC presents significant challenges in accurately predicting VEGF status and patient outcomes.

**WHAT IS NEW HERE**

✓ Using [^18^F] fluorodeoxyglucose positron emission tomography/computed tomography imaging, we developed a noninvasive imaging biomarker capable of predicting both VEGF status and prognosis in patients with GC.
✓ This noninvasive imaging biomarker enables the delivery of precise and personalized treatment for patients.

## Supplementary Material

SUPPLEMENTARY MATERIAL
