# Supplementary material for: Noninvasive Assessment of Vascular Endothelial Growth Factor and Prognosis in Gastric Cancer Through Radiomic Features
Source: Clin Transl Gastroenterol. 2024 Dec 30;16(3):e00802. doi: 10.14309/ctg.0000000000000802 (PMC11932601; doi:10.14309/ctg.0000000000000802)
Supplement: SUPPLEMENTARY MATERIAL [file ct9-16-e00802-s001.docx]

SUPPLEMENTAL FIGURE LEGENDS

**Figure S1. Texture feature selection using the LASSO logistic regression model.**

Notes: (A) The regularization parameter (λ) choice in the LASSO model employed the minimal criterion of 5-fold cross-validation. The Partial Likelihood Deviation (PLD) curve depicts the relationship between log(λ). The vertical dashed line represents the optimal value selected using the minimal criterion and the one standard error (1-SE) standard. The λ chosen through 5-fold cross-validation is 0.05158046, with log(λ) at -1.28751480 (minimal criterion). (B) LASSO coefficient profiles of PET/CT texture features. The coefficient profiles are plotted against the sequence of log(λ). A vertical line is drawn at the selected value found through 5-fold cross-validation, resulting in the optimal λ producing ten non-zero coefficients.

**Figure S2. Performance of the VEGF prediction model.**

Notes: (A-B): ROC curves for the nomination grade, RS, clinical M stage, and location in the Training (A) and Validation (B) groups. (C-D): Calibration curves for RS prediction of VEGF in the Training (C) and Validation (D) groups. (E-F): DCA curves for RS in the Training (E) and Validation (F) cohorts. RS: VEGF Rad score; cM: Clinical M staging.

**Figure S3. Kaplan-Meier analysis of GC patients receiving or not receiving chemotherapy in the training, validation, and testing groups.**

Notes: (A-C): Total survival rates of chemotherapy-naïve GC patients stratified by RS-High and RS-Low in the Training (A), Validation (B), and Testing (C) cohorts. (D-F): Total survival rates of GC patients receiving chemotherapy, derived from RS-High and RS-Low stratifications in the Training (D), Validation (E), and Testing (F) cohorts. Chemo: Chemotherapy.

**Figure S4. Non-invasive biomarker estimation of OS in GC patients.**

Notes: Each variable of the patient is located on a variable-score axis. Draw an upward line on the dot coordinate axis to determine how many points the patient's score contributes to the OS probability. Repeat this process for each variable. Sum the scores of each risk factor. Locate the final total on the total points axis. Draw a line to determine the patient's 1-year, 3-year, or 5-year survival probability. RS: VEGF Rad score; M: Clinical M staging, 1: Metastasis, 0: None.

**Figure S5. Performance of non-invasive imaging biomarkers in predicting OS.**

Notes: (A-C): Time-dependent ROC curves of non-invasive imaging biomarkers predicting OS in the Training (A), Validation (B), and Testing (C) groups. 1-Y: 1-year survival probability; 2-Y: 2-year survival probability; 3-Y: 3-year survival probability.
